# Cross State-dependent Learning Interaction Between Scopolamine and Morphine in Mice: The Role of Dorsal Hippocampus

**DOI:** 10.18869/nirp.bcn.8.3.193

**Published:** 2017

**Authors:** Morteza Maleki, Majid Hassanpour-Ezatti, Majid Navaeian

**Affiliations:** 1. Department of Biology, School of Basic Sciences, Shahed University, Tehran, Iran.; 2. Department of Biology, Shahr Rey Branch, Islamic Azad University, Tehran, Iran.

**Keywords:** Morphine, Scopolamine, CA1 region, Hippocampal, Dependency, Mice

## Abstract

**Introduction:**

The current study aimed at investigating the existence of the cross state-dependent learning between morphine and scopolamine (SCO) in mice by passive avoidance method, pointing to the role of CA1 area.

**Methods:**

The effects of pre-training SCO (0.75, 1.5, and 3 μg, Intra-CA1), or morphine (1, 3, and 6 mg/kg, intraperitoneal (i.p.) was evaluated on the retrieval of passive avoidance learning using step-down task in mice (n=10). Then, the effect of pretest administration of morphine (1.5, 3, and 6 mg/kg, i.p.) was examined on passive avoidance retrieval impairment induced by pre-training SCO (3 μg/mice, Intra-CA1). Next, the effect of pretest Intra-CA1 injection of scopolamine (0.75, 1.5, and 3 μg/mice) was evaluated on morphine (6 mg/kg, i.p.) pre-training deficits in this task in mice.

**Results:**

The pre-training Intra-CA1 injection of scopolamine (1.5 and 3 μg/mouse), or morphine (3 and 6 mg/kg, i.p.) impaired the avoidance memory retrieval when it was tested 24 hours later. Pretest injection of both drugs improved its pre-training impairing effects on mice memory. Moreover, the amnesia induced by the pre-training injections of scopolamine (3 μg/mice) was restored significantly (P<0.01) by pretest injections of morphine (3 and 6 mg/kg, i.p.). Similarly, pretest injection of scopolamine (3 μg/mice) restored amnesia induced by the pre-training injections of morphine (6 mg/kg, i.p.), significantly (P<0.01).

**Conclusion:**

The current study findings indicated a cross state-dependent learning between SCO and morphine at CA1 level. Therefore, it seems that muscarinic and opioid receptors may act reciprocally on modulation of passive avoidance memory retrieval, at the level of dorsal hippocampus, in mice.

## 1. Introduction

Drug-induced state-dependent learning and memory is defined as experiences in which an animal learns skills or relationships between different stimuli under the influence of a psychoactive drug. Memories, stored under drugs influence, are more likely to be performed, recalled, or retrieved under the influence of the same or similar drugs than no or dissimilar drugs (Young, 2014). But, cross state-dependent learning is defined as a situation in which administration of a drug at pretesting periods can restore the amnesia induced by other dissimilar drugs administrated during pre/post-training period and vice versa (Jamali-Raeufy, Nasehi, Ebrahimi-ghiri, & Zarrindast, 2011).

Scopolamine (SCO) is a cholinergic antagonist that blocks most types of muscarinic receptors and induces amnesia via dysregulation of cholinergic system activity (Piri, Rostampour, Nasehi, & Zarrindast, 2013). Scopolamine is also used by scientists to induce memory failure in a variety of learning and memory models, especially in step-down passive avoidance memory in human and mice (Duka, Edelmann, Schutt, Dorow, & Fichte, 1992; Lo et al., 2014). Its effect on the learning, acquisition, and short-term retention of spatial memory tasks is also evaluated (Klinkenberg & Blokland, 2010). It is proposed that SCO memory impairing effect is mediated via encoding disruption in both CA3 and CA1 sub-regions of the hippocampus (Antonova et al., 2011). Its state-dependent memory effect in human and some laboratory animals was also reported previously (Ghorbanalizadeh et al., 2008; Petersen, 1979). Central effects of scopolamine show reversible properties and some neuromodulatory compounds. For example, galantamine can improve its amnesic effect on memory function (Ramakrishnan, Amatya, DeSaer, Dalhoff, & Eggerichs, 2014).

Impairment in passive avoidance memory can be also induced by both pre-training and/or pretest administration of morphine (Zarrindast, Ardjmand, Rezayof, & Ahmadi, 2013). This impairment is reversed by the mu–opioid receptor antagonist naloxone, indicating that mu-opioid receptor plays a role in state-dependent learning of morphine (Jafari-Sabet & Jannat-Dastjerdi, 2009). Patti et al. (2005) denoted that such deficits did not result from other Central Nervous System (CNS) side effects of morphine such as change in anxiety levels or locomotor activity.

On the other hand, an interaction was observed between SCO and morphine effects on different learning and memory tasks such as conditioned place preference in rats (Zhai et al., 2008). The study on genetically manipulated mice showed that muscarinic receptors play important roles in many behavioral effects of morphine (Carrigan & Dykstra, 2007). Moreover, administration of SCO can inhibit parts of morphine withdrawal signs in morphine-dependent rats (Xiang et al., 2006). Although the role of SCO in modulation of stimulation of brain reward system impairment via hippocampus in self-stimulation behavior was less consistent (Vega-Flores, Gruart, & Delgado-García, 2014), it was shown that it can induce recovery in an inhibitory avoidance response after its extinction in rats (Roldán, Cobos-Zapiain, Quirarte, & Prado-Alcalá, 2001).

The dorsal pole of the hippocampus in rodents is known as functional equivalent to the human posterior hippocampus (Fanselow & Dong, 2010). Further, due to its central role in inhibitory avoidance tasks, scientist used direct itra-CA1injection of different compounds in rodents to evaluate different neurotransmitters and their effects upon learning and memory functions (Mahmoodi, Ahmadi, pourmotabbed, Oryan, & Zarrindast, 2010; Zarrindast, Ownegh, Rezayof, & Ownegh, 2014). In this regard, the manipulation of M1 muscarinic receptors on CA1 pyramidal cells proved the participation of dorsal hippocampus muscarinic receptors on learning and memory functions (Leung and Peloquin, 2009). Other researchers also showed the implication of a dorsal hippocampal μ-opioid receptor mechanism in state-dependent memory (Jafari-Sabet & Jannat-Dastjerdi, 2009; Zarrindast, Fazli-Tabaei, Ahmadi, & Yahyavi, 2006a). Moreover, the results of other studies indicate that cholinergic system in this area has critical function in mediating the cross state-dependency of learning and memory (Alijanpour and Rezayof, 2013). Muscarinic receptors have strong modulatory effect on inhibitory synaptic rhythms in hippocampus (Alger, Nagode, & Tang, 2014). Finally, the regulatory role of opioid receptor on muscarinic receptor-mediated synaptic responses in rat hippocampus was reported (Kearns, Morton, Bulters, & Davies, 2001).

Step-down passive avoidance model in rodents is known as a reliable method for pharmacological evaluation of memory and memory-related phenomena such as Long-Term Potentiation (LTP) in CA1 (Whitlock, 2006). The step-down passive avoidance memory in animals relies heavily on the dorsal hippocampus activity (Jafari-Sabet, 2011). Therefore, this method is routinely selected to evaluate hippocampal related state-dependent learning in rodents. The current study aimed at identifying the existence of cross state-dependency of learning between SCO and morphine in step-down type passive avoidance task in mice, considering the role of dorsal hippocampus.

## 2. Methods

### 2.1. Animals and housing conditions

Adult male mice (NMRI species), weighing 20 to 25 g at the time of surgery (Shahed University, Tehran, Iran) were used in the current study. Access to food and water was unrestricted and each 4 animals were housed in a cage. Mice were housed in an animal facility maintained at 22±2°C and 55%±5% relative humidity under a 12:12 hour light/dark cycle with lights on at 7:00 AM. Ten animals were used in each experimental group. Each animal was used once only. All procedures were performed in accordance with the institutional guidelines for animal care and use.

### 2.2. Drugs

Morphine sulfate was purchased from Temad (Tehran, Iran) and was used intraperitoneally (i.p.). Scopolamine hydrochloride and naloxone hydrochloride were purchased from Sigma (St. Louis, MO, USA). Scopolamine hydrobromide was dissolved in sterile saline and injected into CA1 of dorsal hippocampus in a volume of 0.5 μL/site.

### 2.3. Surgery

Mice were anesthetized with a ketamine (50 mg/kg, i.p.)+xylazine (5 mg/kg, i.p.) mixture. Stereotaxic surgery was performed in a standard rodent stereotaxic procedure. Stainless steel guide cannulae (22-gauge) were bilaterally implanted in the CA1 region according to the rat brain atlas of Paxinos and Watson (Paxinos & Franklin, 2001). Guide cannula insertion positions were determined as follows: AP: –2mm from bregma, L: ±1.6 from the sagittal suture, and V: –1.5 mm from the skull surface. The guide cannulae were fixed to the skull by stainless steel skull screws and dental acrylic. Stainless steel stylets (27-gauge; outer diameter: 0.4 mm) were inserted into the guide cannulae to keep them free of debris. All animals were allowed to recover from surgery and clear anesthetic effects for 1 week.

### 2.4. Intra-CA1 injection

During the Intra-CA1 injection, the animals were restrained manually. A 27-gauge injection needle (1 mm below the tip of the guide cannulae) was inserted into the guide cannula. Drugs were delivered manually with a 2.5 μL Hamilton microsyringe attached to the injection cannula via polyethylene tubing (PE-10). The total injection volume was 1 μL per mouse (0.5 μL in each side) over a 60-second period. The injection needle was not removed for an additional 60 seconds to facilitate the diffusion of the drugs (Houghoghi, Rezayof, Zyaian, & Zarrindast, 2009).

### 2.5. Verifying cannula placement

The mice were anesthetized after the testing sessions and 0.5 μL/site of a 4% methylene-blue solution was infused into the CA1. Mice brains were removed after decapitation, and placed in 10% formaldehyde. Then, according to Paxinos and Watson, the selected CA1 areas were sliced and the sites of injections were verified (Paxinos & Franklin, 2001).

### 2.6. Passive avoidance apparatus

Behavioral procedures started 5 to 7 days after the surgery. The apparatus was a wooden box (30×30×40 cm high) and its bottom made of a series of parallel stainless steel bars (0.3 cm diameter spaced 1 cm apart). A wooden platform (4×4×4 cm) was located on the center of the grid floor. The animals were placed on the platform and their latency to step down the grid with 4 paws was measured during the training session. When the animals stepped down, electric shocks (1 Hz, 0.5 s, 45 V DC) were given continuously for 15 seconds. Animals spending more than 20 seconds on the platform or the ones that stepped up the platform before 15 seconds of electric shocks were eliminated from the experiments. Retention test (24 hours after training) was similar to training, but no shock was given to the animals. Latency to step-down was recorded and taken as a measure of memory retention. The upper cut off time was 180 seconds (Rezayof, Amini, Rassouli, & Zarrindast, 2006; Zarrindast and Rezayof, 2004). The retention test was performed between 8:00 AM and 2:00 PM.

### 2.7. Drug treatment

Each experimental group contained 10 mice. In the experimental group, each animal received 2 injections. All control groups received 2 injections of saline.

#### 2.7.1. Evaluation of passive avoidance memory retrieval following pre-training and pretest (Intra-CA1) administration of scopolamine

In the current experiment, 4 groups (10 mice per group) of mice were examined. Control animals received 1 μL/mouse saline solution, whereas other groups were pre-treated with scopolamine (0.75, 1.5, and 3 μg/mouse, Intra-CA1) 5 minutes before training. On the retrieval testing day, the control mice received saline solution (1 μL/mouse) 5 minutes prior to testing. Three other groups received the same doses of scopolamine (0.75, 1.5, and 3 μg/mouse, Intra-CA1) 5 minutes before testing.

#### 2.7.2. Evaluation of passive avoidance memory retrieval following pre-training and pretest intraperitoneally administration of morphine

In this part of the experiment, 40 mice were divided into the following groups: Control group: mice received only vehicles (1 mL/kg); morphine treated groups received different doses (1, 3, and 5 mg/kg, i.p.) of morphine 30 minutes before training. On the test day, control mice received saline (1 mL/kg) 30 minutes prior to testing. But, other groups received different doses of morphine (1, 3, and 5 mg/kg, i.p.) 30 minutes before testing.

#### 2.7.3. Assessment of pretest intraperitoneally morphine injection on memory retrieval impairment induced by Intra-CA1 injection of scopolamine

In this part, mice were divided into 2 control group that received saline (1 mL/mouse, Intra-CA1) before training, and saline (1 μL/mouse, Intra-CA1) 5 minutes before testing, and 3 treatment groups that received pretest morphine (1.5, 3, and 6 mg/kg, i.p.) 5 minutes before testing and pre-training scopolamine (3 μg/mouse, Intra-CA1).

#### 2.7.4. Assessment of pretest (Intra-CA1) administration of scopolamine on morphine induced passive avoidance retrieval impairment

The mice were divided into the following groups: controls that received saline (1 μL/mouse, Intra-CA1) before training and after 24 hours of testing; control mice received saline (1 μL/mouse, Intra-CA1) 5 minutes before the onset of testing. Another 3 groups of animals received morphine (1, 3, and 6 mg/kg, i.p.) pre-training and 24 hours after training, they received saline (1 μL/mouse, Intra-CA1) or scopolamine (3 μg/mouse, Intra-CA1) 5 minutes before the onset of testing.

### 2.8. Data analysis

Data (Step-down latencies) are expressed as mean and interquartile ranges. Large individual variations were observed in the step-down latency time. Data were analyzed with the Kruskal–Wallis nonparametric one-way analysis of variance (ANOVA). Comparisons between the paired groups were performed using the nonparametric Mann– Whitney U-test. For all statistical tests, P<0.05 was statistically significant.

## 3. Results

### 3.1. Effects of pretest Intra-CA1 administration of SCO in mice pre-trained under SCO

**Figure 1.**
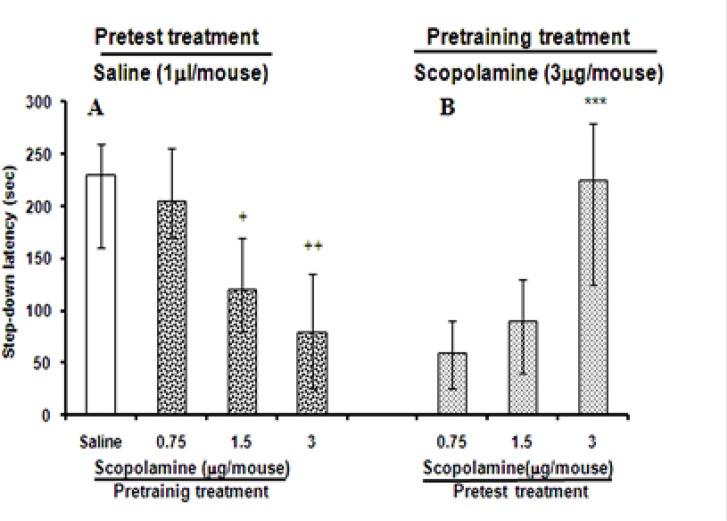
The effects of pre-training and pretest administration of scopolamine on memory retrieval. Different doses of scopolamine (0.75, 1.5, and 3 μg/mouse) and saline (1 μL/mouse) were administered Intra-CA1 30 minutes before training the animals. On the test day, all animals received saline (1 μL/mouse, Intra-CA1) 30 minutes before the test (Panel A). Other groups of animals received pre-training scopolamine (3 μg/mouse, Intra-CA1) and pre-testing injections of different doses of scopolamine (0.75, 1.5, and 3 μg/mouse, Intra-CA1) (Panel B). Test session step-down latencies are expressed as median and quartile for 10 animals. +P<0.05, ++P<0. 01, compared to post-training saline/pretest saline. ***P<0.001, compared to pre-training scopolamine (3 μg/mouse)/pretest saline.

Pre-training administration of different doses of SCO (0.75, 1.5, and 3 mg/mice, Intra-CA1) altered the memory retrieval significantly (the Kruskal–Wallis, non-parametric ANOVA, H (2)=27.18, P<0.01) on the test day, compared with that of saline-treated animals (Figure 1, left panel). In the present experiments, memory retrieval significantly (P<0.05) impaired after the 2 doses of SCO (1.5 and 3 μg/mouse, Intra-CA1) on the test day; although, there was no significant effect of 0.75 μg/mouse SCO on memory retrieval (Figure 1, left panel). The greatest effect was observed at 3 μg/mouse Intra-CA1 of SCO. Administration of SCO (3 μg/mouse, Intra-CA1) prior to training impaired memory retrieval on the test day (Figure 1, right panel), but pretest administered SCO (1.5 and 3 μg/mouse, Intra-CA1) restored it (SCO state-dependent memory) in mice (the Kruskal–Wallis non-parametric ANOVA, H (2)=21.99, P<0.001) (Figure 1, right panel).

### 3.2. Effect of pretest intraperitoneal injection of morphine in mice pre-trained under morphine

**Figure 2.**
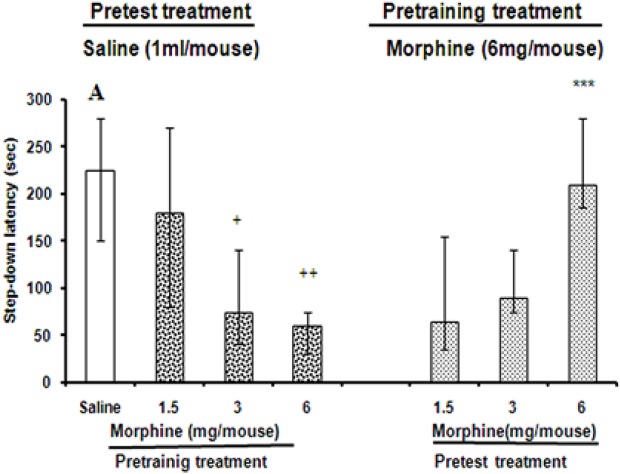
The effects of pre-training and pretest administration of morphine on memory retrieval. Different doses of morphine (1.5, 3, and 6 mg/mouse, i.p.) or saline (1 mL/mouse, i.p., control group) were administered 30 minutes before training the animal groups. On the test day, the control animals received saline (1 mL/mouse, i.p.) 30 minutes before the test (Panel A). Other groups of animals received pre-training morphine (6 mg/mouse, i.p.) and pretesting injections of different doses of morphine (1.5, 3, and 6 mg/mouse, i.p.) (Panel B). Test session step-down latencies are expressed as mean and quartile for 10 animals. +P<0.05, ++P<0.01, compared to pre-training saline/pretest saline. ***P<0.001, compared to pre-training morphine (6 mg/mouse)/pretest saline.

Morphine (1.5, 3, and 6 mg/kg, i.p.) administration prior to the test impaired the memory retrieval in mice pre-trained under morphine, compared with saline-treated animals (Figure 2, right panel). Nevertheless, the high doses of morphine (3 and 6 mg/kg, i.p.) induced significant impairment in memory retrieval on the test day (the Kruskal–Wallis non-parametric ANOVA, H (2)=18.98, P<0.01), the low dose of morphine (1.5 mg/kg, i.p.) had no significant effect on memory retrieval. The maximum response was obtained with 6 mg/kg of drug. Pre-training administration of morphine (6 mg/Kg, i.p.) impaired memory retrieval on the test day (Figure 2, left panel), but it was restored when morphine (6 mg/kg, i.p.) was administered as pretest treatment (morphine state-dependent memory) in mice (the Kruskal–Wallis, non-parametric ANOVA, H (2)=27.18, P<0.01).

### 3.3. Effect of pretest administration of SCO on memory impairment induced by pretraining morphine and pretest morphine administration on pretraining SCO induced amnesia; a cross state dependency

**Figure 3.**
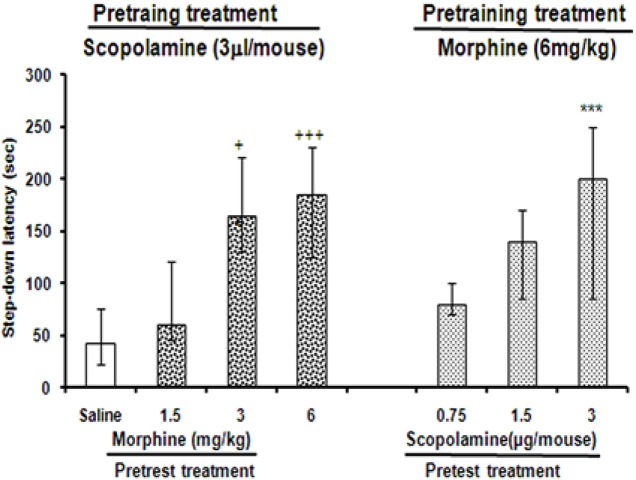
Cross-state dependent interactions between scopolamine and morphine in the passive avoidance memory task in mice. The improving effects of pretest intraperitoneal morphine administration on passive avoidance impairment induced by pre-training Intra-CA1 scopolamine (right). Pretest Intra-CA1 administration of scopolamine improved morphine induced impairment in passive avoidance response of mice (left panel). In the right panel, animals received saline (1 μL/mouse, Intra-CA1 and control) or scopolamine (3 μg/mouse, Intra-CA1) 30 minutes before training. The animals were tested after Intra-CA1 administration of morphine (1.5, 3, and 6 mg/mouse) on the test day. Animals in the left panel received saline (i.p.) or morphine (6 mg/mouse, i.p.) before training (panel B). On the test day, all animals in the left panel were tested 30 minutes after Intra-CA1 administration of scopolamine (0.75, 1.5, and 3 μg/mouse). Test session step-down latencies are expressed as mean and quartile for 10 animals. +P<0.05, +++P<0.001 compared to pre-training saline/pretest saline. ***P<0.001, compared to pre-training morphine/pretest saline.

Pretest application of morphine (3 and 6 mg/kg, i.p.) could significantly (the Kruskal–Wallis nonparametric ANOVA, H (6)=121.34, P<0.05 and P<0.001, respectively) reverse the pertaining Intra-CA1 amnesic effect of SCO (3 μg/rat) (Figure 3, left panel). Similarly, pretest administration of SCO (3 μg/mice, Intra-CA1) reversed (the Kruskal–Wallis ANOVA, H (6)=31.20, P<0.001) the impairment of retention induced by pre-training injection morphine (6 mg/kg, i.p.) in mice (Figure 3, right panel).

## 4. Discussion

The results of the current study showed that the pre-training Intra-CA1 injection of SCO could impair the memory retrieval of mice when tested 24 hours later in step-down passive avoidance task. The current study findings were in agreement with those of similar observations by Piri et al. (2013). They showed that pre-training Intra-CA1 administration of SCO could induce memory impairment in one-trial step-down inhibitory avoidance task in rats. In this respect, the essential role of dorsal hippocampus cholinergic afferents was shown when both acquisition and retention of avoidance learning were controlled in rodents (Hung, Lin, Liao, & Wang, 2004). Also, studies on genetically muscarinic receptors knock-out mice showed that different subtypes of muscarinic receptors participated in the regulation of normal hippocampal cognitive functions (Tzavara et al., 2003).

The microdialysis studies on rats showed that release of acetylcholine developed from hippocampal nerve terminals after administration of SCO (Hiramatsu, Murasawa, Nabeshima, & Kameyama, 1998). This enhanced release of acetylcholine may be caused by positive feedback after the blockade of presynaptic autoreceptors and/or post-synaptic muscarinic receptors (Hiramatsu et al., 1998). Thus, acetylcholine release in dorsal hippocampus during pretest may provide a base to overcome the pre-training amnesic effect of muscarinic blockage on passive avoidance memory (Souza, Bruning, Acker, Neto, & Nogueira, 2013, Mitsushima, Sano, & Takahashi, 2013).

The current study findings about memory impairing effects of morphine after pre-training administration were also supported by previous research. For example, Zarrindast et al., suggested that intraperitoneal pre-training administration of morphine to mice impaired memory formation in the step-down experiment (Zarrindast, Kangarlu-Haghighi, Khalilzadeh, & Fazli-Tabaei, 2006b). Such effects were mediated only via the activation of mu-opioid receptor (Shiigi, Takahashi, & Kaneto, 1990), and high levels of mu-opioid receptors were expressed in the hippocampal formation (De Oliveira et al., 2010). In addition, the role of hippocampus mu-opioid receptors in memory was recently confirmed by studying gene knock-out systems in mouse (Jang et al., 2003).

Finally, cross state-dependent effect was observed between morphine and SCO on retrieval of passive avoidance memories in mice. It seems that the bidirectional modulatory relationship between the cholinergic and opioids systems in hippocampus provide a base for such cross state-dependent effects in rodents (Zarrindast, Farahmandfar, Rostami, & Rezayof, 2006c). One suggested mechanism for explanation of cross state-dependent effect between morphine and scopolamine is that each system can simulate the effects of other systems on CA1 neurons or circuits. Previous studies on chick brain demonstrated that the mu-opioid receptors on cholinergic terminals in the hippocampus were normally under tonic inhibition by the endogenous opiate system (He, Chen, Wang, & Ma, 2008).

The intracerebroventricular administration of a low dose of endomorphin-1 and an endogenous mu-opioid receptor agonist could improve the SCO-induced impairment in a short-term memory task in mice (Sakaguchi Koseki, Wakamatsu, & Matsumura, 2003). On the other hand, morphine could increase acetylcholine release from brainstem synapses by synaptic disinhibition (Zhu, Bowman, Baghdoyan, & Lydic, 2008). Altogether, it seems that a bidirectional relationship links the opioid and cholinergic systems at hippocampal level. Li et al., using the schedule-controlled responding technique in rats investigated the existence of a direct functional and neurochemical interaction between the mu-opioid receptor agonist morphine and the muscarinic cholinergic antagonist scopolamine (Li et al. 2010). Their results confirmed the functional bidirectional interaction between morphine and scopolamine for CA1-related memory functions (Nasehi et al., 2014).

In an electrophysiological study, Kearns et al. (2001) showed the mu-opioid receptor-mediated presynaptic enhancement of muscarinic receptor mediated EPSPs in single hippocampal CA1 pyramidal neurons. Indeed, both scopolamine and morphine are alkaloid and show many functional and pharmacological similarities at intracellular level and their signaling cascade (Telegdy, Bagosi, & Jaszberenyi, 2014). Altogether, it seems that interaction between SCO and morphine can activate the same mechanisms in CA1 area and provide a basis for cross state-dependent learning mechanism.

Another hypothesis to explain the cross state-dependent learning phenomenon between SCO and morphine is that these compounds can indirectly simulate the effects of each other on passive avoidance via modulation of GABAergic system at CA1 level. Studies showed that muscarinic receptor antagonists impair memory by indirect modulation of inhibitory GABAergic interneuron in hippocampus (Chang & Liang, 2012). Also, it is shown that mu-opioid receptor modulate GABA (γ-Aminobutyric acid) receptors in hippocampal CA1 synapses (McQuiston, 2007). Hippocampal gene expression profile also provided further evidence for possible involvement of GABA receptors in SCO-induced amnesia (Brouillette, Young, During, & Quirion, 2007).

The results of electrophysiological studies also confirmed that specific classes of GABAergic interneurons are the main sources of endogenous opioids and muscarinic receptors in the hippocampus that innervate other types of interneurons (Giannopoulos & Papatheodoropoulos, 2007; Fukudom et al., 2004). Interconnections between cholinergic, opioidergic, and GABAergic neurotransmission are reported at CA1 region of guinea pigs (Favaroni Mendes & Menescal-de-Oliveira, 2008). Parsaei et al., also showed the role of GABAergic system of hippocampus in mediating the state-dependent learning and memory (Parsaei, Rangchiyan, Ahmadi, & Zarrindast, 2011). Therefore, it seems that GABAergic system of CA1 area can act as an interface between opioid and cholinergic systems in cross state-dependent learning (Rezayof, Razavi, Haeri-Rohani, Rassouli, & Zarrindast, 2007). Thus, a second suggested hypothesis is the existence of an indirect interaction between cholinergic and opioidergic systems at the level of CA1, which states that GABAergic interneurons can act as intermediate pathway for cross state-dependent learning between morphine and SCO.

In summary, results of the current study showed that pretest intra CA1 injection of SCO fully reversed pre-training morphine-induced amnesia in step-down passive avoidance in mice and vice versa, which indicated the existence of cross state-dependency learning between morphine and SCO. To explain the findings, the following hypotheses are suggested: (i) SCO and morphine can affect and simulate the functions of each other via bidirectional interaction in CA1 area or (ii) SCO and morphine can simulate the effects of each other by indirect interactions via GABAergic system, and hence, shape a cross state-dependent memory effect between each other.
